# Effectiveness of modified Valsalva maneuver by using wide bore syringe for emergency treatment of supraventricular tachycardias: Findings from Pakistan

**DOI:** 10.12669/pjms.39.3.6725

**Published:** 2023

**Authors:** Hira Ashraf, Turab Fatima, Ifra Ashraf, Sadaf Majeed

**Affiliations:** 1 Dr. Hira Ashraf, FCPS. Department of Emergency Medicine, Pakistan Ordinance Factory Hospital, Wah Cantt, Pakistan; 2 Dr. Turab Fatima, FCPS. Department of Emergency Medicine, Pakistan Ordinance Factory Hospital, Wah Cantt, Pakistan; 3 Dr. Ifra Ashraf, FCPS. Department of Physiology, Shifa College of Medicine, Islamabad, Pakistan; 4 Dr. Sadaf Majeed, FCPS. Department of Physiology, Shifa College of Medicine, Islamabad, Pakistan

**Keywords:** Modified Valsalva maneuver, Supraventricular tachycardia, Efficacy

## Abstract

**Background and Objectives:**

The Valsalva maneuver (VM) is the most effective measure that can be carried out to treat supraventricular tachycardia (SVT). Our objective was to compare the efficacy of postural modified VM with 20 ml syringe to standard VM for the emergency treatment of SVT.

**Methods:**

This randomized control trial study was conducted at the Accident and Emergency Department, Pakistan ordinance factories hospital, Wah Cantt from July 2019 to September 2020. In the standard Valsalva group, fifty patients were placed at an angle of 45 with continuous monitoring of vitals and electrocardiogram. Patients blew into a 20ml syringe to generate 40 mmHg pressure for 15 seconds and remained in the same position for 45 seconds before a reassessment of cardiac rhythm at one-minute and three-minute intervals. In the modified Valsalva group same procedure was repeated with the other fifty patients, but immediately at the end of the strain, they were laid flat with their legs raised to 45° for 15 seconds. Participants returned to semi-recumbent position and cardiac rhythm was reassessed after 45 seconds and then at one and three minutes.

**Results:**

In the standard Valsalva maneuver (SVM)20.0% of participants versus 58% of participants in the modified Valsalva maneuvers group(MVM) reverted to sinus rhythm at one min (odds ratio or 5.52, 95% CI 2.26-13.47; p<0.001) and time of stay in the emergency room was (odds ratio or 2.39, 95% CI 1.45- 3.93; p<0.0001).

**Conclusion:**

Modified Valsalva by using a wide-bore syringe is more effective method than standard Valsalva in terminating SVT.

## INTRODUCTION

Supraventricular tachycardia (SVT) is a collection of cardiac arrhythmia that encompass recurrent episodes of regular narrow QRS complex tachycardia and irregular narrow QRS complex tachycardia.^1^,^2^ Regular, narrow complex tachycardia involve atrioventricular nodal reentrant tachycardia, atrioventricular reentrant tachycardia, atrial tachycardia (AT), sinuses tachycardia. Irregular, narrow complex tachycardia consists of atrial fibrillation, multifocal AT, and atrial flutter with variable block.^3^

There are numerous types of symptoms accompanying with SVT, starting from nearly asymptomatic to having signs and symptoms of low cardiac output, syncope, palpitations, fatigue, lightheadedness, chest discomfort or dyspnea. The SVT episodes not only occur in the condition of emotional stress, but can also occur at rest.^4^ The 12-lead ECG is specially favored for analysis of SVT, helps in determining that the affected person is suffering from which sort of SVT. After taking ECG recordings in hemodynamically stable patients, nonpharmacological approach like vagal maneuver is recommended under continuous ECG monitoring. By use of this maneuver specific patients who respond to the vagal maneuver (VM) may be identified and these patients can gain knowledge about how to apply it by themselves in future episodes.^5^

Vagal maneuvers are procedures used to increase vagal parasympathetic tone with the intention to diagnose and deal with numerous arrhythmias. The most commonly used VM are carotid sinus massage, Valsalva maneuver and diving reflex.^6^ The VM certainly, considered as one of the vagal maneuvers, was first identified in 1704, includes expiring against a closed glottis to increase intrathoracic pressure up to 40 mmHg, stimulating baroreceptor activity and increased vagal tone.^7^ When efficiently performed, the success rate of this maneuvers in terminating SVT has been reported as between 19 and 54%.

The VM is a safe and across the world encouraged first-line emergency treatment for SVT. It is usually proven to be most effective in adults. Blowing into the syringe with forced expiration at the same time as the glottis is closed is a simple method for performing VM while patient lying down having face up for 15 seconds. This generates increased pressure within the chest cavity and triggers a slowing of heart rate that could prevent abnormal rhythm.

Other techniques like placing a thumb on the mouth and blowing against it, sitting down and pushing against the hand on the abdomen.^8^ VM with modifications makes it more effective in terminating SVT. These modifications include pressing the epigastric zone or abdomen for 10 seconds after VM, changing position quickly such as from being seated to supine or lifting the feet at a 45-degree angle just after the VM or blowing into different size syringes to generate recommended intrathoracic pressure. MVM technique like involving a passive leg elevation immediately after straining has newly revealed more success rate by increasing the relaxation phase of venous return and vagal stimulation than any other method. 7,9,10

To the best of our knowledge, only a few studies had evaluated the effectiveness of a modified VM compared with the standard VM in Pakistani patients with SVT. Therefore, in this study, we aimed to evaluate the clinical efficacy and economic efficiency, as well as the safety of a modified VM vs standard VM in Pakistani patients with SVT. This method can also explain to the patient so that they can use it by their own during an episode of SVT. This will be beneficial not only for patients but also for health-care providers worldwide, including regions with few health-care resources.

## METHODS

After calculating sample size in the randomized control trial study done during July 2019 to September 2020 in Accident and Emergency Department, Pakistan ordinance factories hospital, Wah Cantt through WHO sample size calculator was used to calculate the sample size. In this population proportion of sinus rhythm achieved in standard Valsalva group = 0.17 and population proportion of sinus rhythm achieved in modified Valsalva group = 0.43.^9^ Power of test 80%, Level of significance 5% and sample size is 50 in each group. By convenience sampling one hundred participants were randomized as 1:1 ratio to be included either in a standardized Valsalva group or a modified Valsalva group after taking approval from the Institutional Review Board (IRB# 4119/HOD ER/HOSP) and informed consent. Patients with an age greater than 18 years and less than 65 in both sexes with supraventricular tachycardia (regular, narrow-complex tachycardia with QRS duration 0.12 seconds on ECG) were included in the study. Unstable patients with systolic blood pressure less than 90 mm Hg or having atrial fibrillation, flutter, acute pulmonary edema, aortic stenosis, recent myocardial infarction, glaucoma, retinopathy, and patients with third trimester pregnancy were not included in the study.

In the standard Valsalva group, patients were placed on a couch at an angle of 45 with continuous vitals and 3-lead ECG monitoring. A common practice to gain prerequisite for performing VM is to request the patient to blow hard into the syringe to move the plunger. Each syringe is opened from its sealed packet and the pressure is released by moving the plunger manually up to ¼ of the volume of the syringe meaning 5mL out of 20mL syringe. In this situation much less straining pressure and exertion is required by the patient and make it easy for them to achieve recommended 40 mg pressure without much effort because as the diameter of the syringe increases it offers less resistance. Finally, the plunger was moved back to the zero mark and then the patient blew into a 20ml syringe to move the plunger to generate 40mmHg pressure for 15 seconds, remained in the same position for 45 seconds. Cardiac rhythm was reassessed by 3-lead ECG at one minute and then at three minute intervals. In the modified Valsalva group, the same procedure was repeated by patient but immediately at the end of the strain, they were laid flat with their legs raised to 45° for 15 seconds. The participant returned to the semi-recumbent position and cardiac rhythm was reassessed after 45 seconds and then at interval of one and three minutes.^7^,^11^,^12^

## RESULTS

Demographic characteristics of both the groups are presented in Table-I and were similar in both groups. The baseline, past medical history, and presenting physiological data are presented in Table-II. The mean values of systolic blood pressure, Diasystolic blood pressure, and oxygen saturation, heart rate at the start and at the end for the MVM group were 125.40±19.29, 82.00±13.60, 98.18±1.11, 185.70±17.41 and 96.06±12.59 respectively. Lesser mean values were observed 122.50±19.54, 83.76±16.43, 98.16±1.67, 184.94±18.20 and 96.98±13.58 respectively in the MVM group. Comparison of reversion of rhythm, time of stay in the emergency room and need for rescue therapy between the two groups are presented in Table-III.

**Table-I:** Analysis of demographic characteristics of standard and modified Valsalva group.

Valsalva Maneuver group	Standard	Modified	p
Statistics	n (%)	n (%)	
Age	50.66±11.58	46.48±10.81	0.065*
Gender			0.688
Male	22(44.0%)	24(48.0%)	
Female	28(56.0%)	26(52.0%)	

* Data was presented in Mean±SD; p-value was obtained from t-test.

**Table-II:** Comparison of baseline characteristics among two group.

Valsalva Maneuver Group	Standard	Modified	

Statistics	n (%)	n (%)	P value
Systolic Blood Pressure	125.40±19.29	122.50±19.54	0.457
Diastolic Blood Pressure	82.00±13.60	83.76±16.43	0.561
Oxygen saturation	98.18±1.11	98.16±1.67	0.944
Heart rate at start	185.70±17.41	184.94±18.20	0.831
Heart rate at end	96.06±12.59	96.98±13.58	0.726
Past SVT diagnosed	17(34.0%)	24(48.0%)	0.155
Ablation therapy	1(2.0%)	3(6.0%)	0.307
Diabetes Mellitus	10(20.0%)	8(16.0%)	0.603
Hypertension	18(36.0%)	16(32.0%)	0.673
Ischemic heart diseases	7(14.0%)	11(22.0%)	0.298
Smoking	12(24.0%)	12(24.0%)	
Asthma/ COPD	6(12.0%)	9(18.0%)	0.401
Valvular heart disease	0(0.0%)	2(4.0%)	0.153

*Data was presented in Mean±SD; p-value was obtained from t-test.

**Table-III:** Comparison of reversion of rhythm and need of rescue therapy among two groups.

Valsalva Maneuver group	Standard	Modified	p
Statistics	n (%)	n (%)	
Rhythm reverted	10(20.0%)	29(58.0%)	<0.001*
Rescue therapy	48(96.0%)	21(42.0%)	<0.001*
Stay in emergency room (hr.)	2.20±0.77	1.53±0.95	<0.001*

* Data was presented in Mean±SD; p-value was obtained from t-test.

Ten (20.0%) of 50 participants in the SVM versus 29 (58%) of 50 participants in the MVM group achieved the outcome as sinus rhythm reverted at one minute (odds ratio [OR] 5.52, 95% CI 2.26–13.47; p<0.001). About 42% of patients treated with modified Valsalva and 80% of those treated with standard Valsalva needed rescue therapy. Time of stay in emergency room in hours was (odds ratio [OR] 2.39, 95% CI 1.45- 3.93; p<0.0001) and median length of stay in hospital was of 2 h (IQR, 2–3) for the standard VM group and 1h (IQR, 1–2) and P = <0.001 was significant for the modified VM group.

## DISCUSSION

Present study has shown that a simple, cost-free, self-applicable MVM is very effective as it reverts 58% of patients to sinus rhythm compared with 20% with an SVM. A study from Pakistan by Malik S et al and Satti KN et al found that the standard Valsalva maneuver was the most common way to stop SVT. According to their study, the success rate of the Valsalva maneuver in reversion was 23% and 27% respectively.^13^,^14^ This difference in success rate may be due to various factors like low understanding of the patient’s ability to perform maneuvers effectively, or the patient may be unable to follow instructions properly. Some other studies from Pakistan by Awan RA et al, Shafquat A et al, and Shaikh SA et al showed that SVT due to any cause can be effectively treated by using radio frequency ablations nevertheless this invasive method required technical facilities and skill expertise which are not readily available in health care system of Pakistan, so by using MVM as used in our study SVT can be treated without involvement of surgical equipment and skills.^15^–^17^

Regional studies by Huang EP et al in 2022, Lan Q et al in 2021and Abdulhamid AS et al in 2021 reported that by using MVM, the conversion rate of SVT to sinus rhythm was more effective than SVM showing that the result of our studies are consistent with their results. They also stated that use of MVM also reduced the use of anti-arrhythmic drugs, stay time in emergency department and adverse events.^18^–^20^ In other two studies conducted by Appelboam et al. and Chen et al showed that there was no significant difference between the time of stay in the hospital in both standard and modified Valsalva maneuver.^9^,^10^ In our study time of stay in the hospital was not significant for the MVM group.

Ekinci et al. in their study performed four different Valsalva maneuver methods consisting of the generation of different straining pressures during different time periods, like 40 mm Hg for 10 seconds, 40 mm Hg for 15 seconds, 50 mm Hg for 10 seconds, and 50 mm Hg for 15 seconds while lying in a supine position. Straining pressure was measured by a sphygmomanometer. The result showed that there were no significant differences in the decreasing heart rates among the four techniques. In addition, there were no differences between the vagal responses in terms of age, gender, and body mass index.^21^ In our study, we used the SVM in patients laying on a trolley at an angle of 45 degrees. Then the patient blew into a 20ml syringe to move the plunger to generate 40mmHg pressure instead of a sphygmomanometer for 15seconds and remained in the same position for 45 seconds before reassessment of cardiac rhythm, initially at one-min and then at three- minutes intervals, the result shows that only 4% of patients showed successful termination of SVT, which is not statistically significant. Similarly, there were no differences between the vagal responses in terms of age, gender, and having a history of previous episodes of SVT, hypertension, diabetes mellitus, ischemic heart disease, and respiratory disorders, as proved by Ekinci et al.

Appelboam et al and Walker et al have claimed that the Trendelenburg posture labelled as modified Valsalva gives a higher number of successful terminations of SVTs, so the “standard technique” is compared with different methods consisting of many different postures and durations.^21^-^23^ The findings of our studies also coincide with the findings of their studies which is that the MVM is more successful than the standard Valsalva. Ferreira et al. and Wheeler et al. conducted studies to compare the MVM with the SVM showing that there was an increase in the rate of termination of SVT to normal sinus rhythm using the modified Valsalva just as in our studies.^24^,^25^ Thornton et al compared different syringes each of 5ml, 10ml, and 20ml using a manual sphygmomanometer. They observed that every syringe requires very high pressure if the plunger has not been previously moved. Once the plunger has been moved, the larger the syringe, the lower the pressure required for the patient to move the plunger and attain straining pressure.^7^

In our study, a 20 ml syringe was used to build a 40 mm Hg pressure. The larger diameter of the syringe helps to establish the required pressure with little effort, which makes it easy to perform the Valsalva maneuver on the patient. Appelboam et al., Çorbacıoğlu et al., and Wang et al. investigated the percentage of SVT reversion with standard and modified Valsalva maneuvers. Appelboam et al. conducted a study showing that 17% of participants assigned to perform the SVM achieve sinus rhythm as compared to 43% of participants performing the MVM. Çorbacıoğlu et al. revealed that 10.7% of the SVM group and 42.9% of the MVM group returned to sinus rhythm. Wang et al. compared the success rates between the two methods after single and multiple sessions. Patient treated with MVM had higher success rates of reversion of SVT after single secession was 47.78% and after multiple secession it was 62.22%, while 15.38% and 19.78% were successful in achieving sinus rhythm after single and multiple secession of the standard method.^9^,^26^,^27^ This appears to be more cost-effective and reduce the financial burden in developing countries not only on patient but also on hospital managment, as it can be adapted by patients for use at home, reduce presentations to hospital and decrease drug need and costs.

### Limitation:

Sample size was not sufficient and can be increased by conducting this study in multiple healthcare centers. There was also a language barrier and the inability of the patient to understand and follow commands for performing maneuver correctly.

## CONCLUSION

The present study’s results suggest that modified VM therapy was more effective than standard VM for terminating SVT. This maneuver should be used in an emergency as it is simple, safe, without any adverse effects, cost-effective, and noninvasive, and it can prevent many patients from being treated with drugs or even seeking treatment from healthcare professionals as the patients can perform this by themselves.

### Author’s Contribution:

**HA, TF, IA** conceived, designed and collect data

**IA, HA** did statistical analysis and manuscript writing

**SM, TF** editing of manuscript and final approval.

**IA** takes the responsibility and is accountable for all aspects of the work in ensuring that questions related to the accuracy or integrity of any part of the work are appropriately investigated and resolved.
